# Supplementary material for: Definition of the Binding Architecture to a Target Promoter of HP1043, the Essential Master Regulator of Helicobacter pylori
Source: Int J Mol Sci. 2021 Jul 22;22(15):7848. doi: 10.3390/ijms22157848 (PMC8345958; doi:10.3390/ijms22157848)
Supplement: Supplementary file 1 [file ijms-22-07848-s001.zip › ijms-1294759-supplementary.pdf]

Supplementary Materials

Definition of the binding architecture of the HP1043 regulatory protein of *Helicobacter pylori* to a target promoter

Annamaria Zannoni [1], Simone Pelliciari [1], Francesco Musiani [1], Federica Chiappori [2], Davide Roncarati [1,*] and Vincenzo Scarlato [1,*]

Table of contents | Pag.

Figure S1: Methyl green did not interfere with HP1043 binding to P_{hp1227} target 2
Figure S2: Molecular dynamics conformations clustering 2
Figure S3: Calculated distance between selected residues at the HP1043-DNA interface 2
Table S1: List of oligonucleotides used in this study 3
Table S2: List of plasmids used in this study 5
Table S3: Binding energy obtained from alanine scanning 7

Figure S1

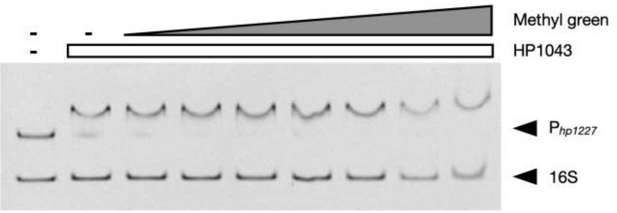

Figure S1: Methyl green did not interfere with HP1043 binding to P_{hp1227} target. EMSA in the presence of a fixed amount of HP1043 (8 µM) and increasing concentrations of the DNA major groove binder methyl green (0.001, 0.01, 0.1, 1, 10, 100, 500 µM). A 60 bp probe of the 16S rRNA gene was used as internal specificity control. Symbols are as in Figure 3.

Figure S2

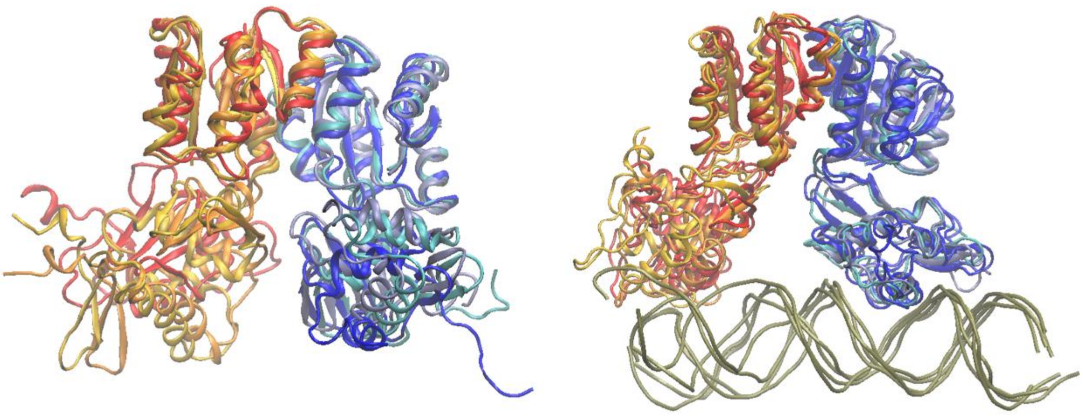

Figure S2: Molecular dynamics conformations clustering. On the left, the unbound HP1043, on the right, the HP1043-DNA bound complex. Chain A is depicted in red, chain B in blue, DNA in green.

Figure S3

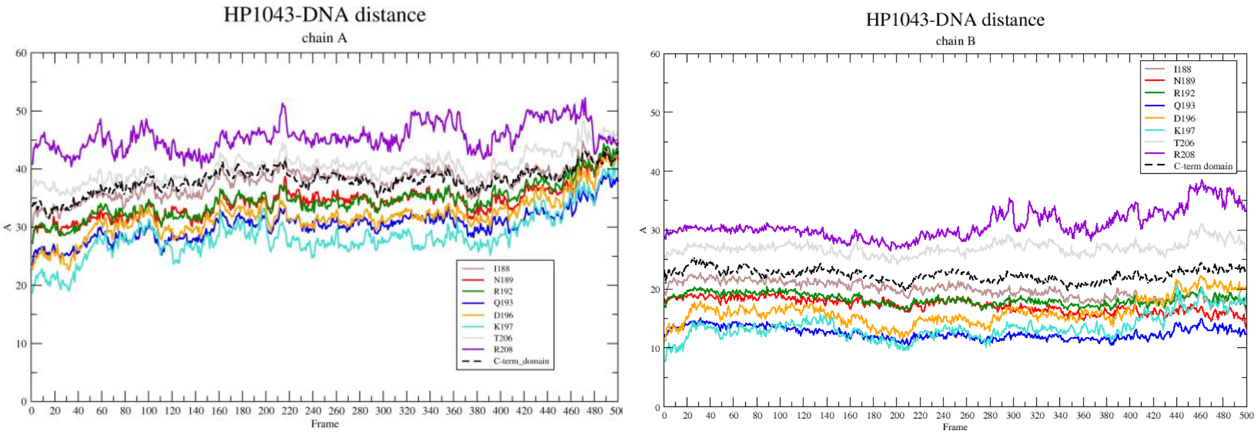

Figure S3: Calculated distance between selected residues at the HP1043-DNA interface. Distance between DBD residues and DNA centers of motion are plotted along the trajectory. The black dashed line shows the distance from each DBD center of motion and the DNA center of motion.

Table S1 - List of oligonucleotides used in this study

Oligo	Sequence (5’ to 3’)	*
p1227WT_F	TTCTCTTATTTTCACTTCATTTTTTAAGCAAAACTTAAACTTGTAATTGTATCATTTTAAG	P
p1227WT_R	CTTAAAATGATACAATTACAAGTTTAAGTTTTGCTTAAAAAATGAAGTGAAAATAAGAGAA	P
p1227T1F_F	TTCTCTTATTTTCACTTCATTTATTAAGCAAAACTTAAACTTGTAATTGTATCATTTTAAG	P
p1227T1F_R	CTTAAAATGATACAATTACAAGTTTAAGTTTTGCTTAATAAATGAAGTGAAAATAAGAGAA	P
p1227T2F_F	TTCTCTTATTTTCACTTCATTTTATAAGCAAAACTTAAACTTGTAATTGTATCATTTTAAG	P
p1227T2F_R	CTTAAAATGATACAATTACAAGTTTAAGTTTTGCTTATAAAATGAAGTGAAAATAAGAGAA	P
p1227T3F_F	TTCTCTTATTTTCACTTCATTTTTAAAGCAAAACTTAAACTTGTAATTGTATCATTTTAAG	P
p1227T3F_R	CTTAAAATGATACAATTACAAGTTTAAGTTTTGCTTTAAAAATGAAGTGAAAATAAGAGAA	P
p1227A1F_F	TTCTCTTATTTTCACTTCATTTTTTTAGCAAAACTTAAACTTGTAATTGTATCATTTTAAG	P
p1227A1F_R	CTTAAAATGATACAATTACAAGTTTAAGTTTTGCTAAAAAAATGAAGTGAAAATAAGAGAA	P
p1227A2F_F	TTCTCTTATTTTCACTTCATTTTTTATGCAAAACTTAAACTTGTAATTGTATCATTTTAAG	P
p1227A2F_R	CTTAAAATGATACAATTACAAGTTTAAGTTTTGCATAAAAAATGAAGTGAAAATAAGAGAA	P
p1227GF_F	TTCTCTTATTTTCACTTCATTTTTTAACCAAAACTTAAACTTGTAATTGTATCATTTTAAG	P
p1227GF_R	CTTAAAATGATACAATTACAAGTTTAAGTTTTGGTTAAAAAATGAAGTGAAAATAAGAGAA	P
p1227ΔF_F	TTCTCTTATTTTCACTTCATTTGGATCCCAAAACTTAAACTTGTAATTGTATCATTTTAAG	P
p1227ΔF_R	CTTAAAATGATACAATTACAAGTTTAAGTTTTGGGATCCAAATGAAGTGAAAATAAGAGAA	P
p1227T1S_F	TTCTCTTATTTTCACTTCATTTTTTAAGCAAAAGTTAAACTTGTAATTGTATCATTTTAAG	P
p1227T1S_R	CTTAAAATGATACAATTACAAGTTTAACTTTTGCTTAAAAAATGAAGTGAAAATAAGAGAA	P
p1227T2S_F	TTCTCTTATTTTCACTTCATTTTTTAAGCAAAACATAAACTTGTAATTGTATCATTTTAAG	P
p1227T2S_R	CTTAAAATGATACAATTACAAGTTTATGTTTTGCTTAAAAAATGAAGTGAAAATAAGAGAA	P
p1227T3S_F	TTCTCTTATTTTCACTTCATTTTTTAAGCAAAACTAAAACTTGTAATTGTATCATTTTAAG	P
p1227T3S_R	CTTAAAATGATACAATTACAAGTTTTAGTTTTGCTTAAAAAATGAAGTGAAAATAAGAGAA	P
p1227A1S_F	TTCTCTTATTTTCACTTCATTTTTTAAGCAAAACTTTAACTTGTAATTGTATCATTTTAAG	P
p1227A1S_R	CTTAAAATGATACAATTACAAGTTAAAGTTTTGCTTAAAAAATGAAGTGAAAATAAGAGAA	P
p1227A2S_F	TTCTCTTATTTTCACTTCATTTTTTAAGCAAAACTTATACTTGTAATTGTATCATTTTAAG	P
p1227A2S_R	CTTAAAATGATACAATTACAAGTATAAGTTTTGCTTAAAAAATGAAGTGAAAATAAGAGAA	P
p1227GS_F	TTCTCTTATTTTCACTTCATTTTTTAAGCAAAACTTAATCTTGTAATTGTATCATTTTAAG	P
p1227GS_R	CTTAAAATGATACAATTACAAGATTAAGTTTTGCTTAAAAAATGAAGTGAAAATAAGAGAA	P
p1227ΔS_F	TTCTCTTATTTTCACTTCATTTTTTAAGCAAAAGGATCCCTTGTAATTGTATCATTTTAAG	P
p1227ΔS_R	CTTAAAATGATACAATTACAAGGGATCCTTTTGCTTAAAAAATGAAGTGAAAATAAGAGAA	P
LuxRT_F	TTGGCAGATGTGTGTACCTTC	RT
LuxRT_R	TGATGACTCCCAAGGAAAAATAG	RT
p1227EcoRV_F	TTCTCTTATTTTCACTTCATTT	C
p1227EcoRV_R	ATATGAATTCCTTAAAATGATACAATTACAAG	C
HP1043_I184A_F	GGCTGAAGTGGCTATCAATCAAATCCG	M
HP1043_E185A_F	GATTGCAGTGGCTATCAATCAAATCCG	M
HP1043_I188A_F	GATTGAAGTGGCTGCCAATCAAATCCGCC	M
HP1043_N189A_F	GATTGAAGTGGCTATCGCTCAAATCCGCC	M
HP1043_N189A_R	ACATTAGGGGTAACCATTTCAGG	M
HP1043_R192A_F	CGCCCAAAAAAATGGATAAACCCTTGG	M
HP1043_R192A_R	ATTTGATTGATAGCCACTTCAATC	M
HP1043_Q193A_F	CCGCGCAAAAATGGATAAACCCTTGGGG	M
HP1043_D196A_F	GCTAAACCCTTGGGGATTTCCACG	M
HP1043_D196A_R	CATTTTTTGGCGGATTTGATTGATAG	M
HP1043_K197A_F	GATGCACCCTTGGGGATTTCCAC	M
HP1043_Y206A_F	GGTTGAAGCTGTAAGGCGCAGAGG	M
HP1043_Y206A_R	GTGGAAATCCCCAAGGGTTTATC	M
HP1043_R208A_F	GGTTGAAACCGTAGCGCGCAGAGGCTATC	M
HP1043_EAA_F	GAAGCTGCAGGGAAGCCTTTTGAAGTGCTTAC	M
HP1043_AAE_F	GCTGCTGAAGGGAAGCCTTTTGAAGTGCTTAC	M
HP1043_EVK_R	AACTTCACGCCCCTTGTAAATAATC	M
HP1043_Q190A_F	AATCCGCCAAAAAATGGATAAAC	M
HP1043_Q190A_R	GCATTGATAGCCACTTCAATCAC	M
HP1043_K194A_F	GCAATGGATAAACCCTTGGGG	M

HP1043_K194A_R	TTGGCGGATTTGATTGATAGC	M
HP1043_R209A_F	GCCAGAGGCTATCGTTTTTGC	M
HP1043_R209A_R	CCTTACGGTTTCAACCGTG	M
HP1043_R210A_F	CGCGCAGGCTATCGTTTTTGCTAC	M
HP1043_CTD_NheI_F	ATATGCTAGCAATGTGATTGAAATTGGGGATTTGAC	C
HP1043_BamHI_R	ATATGGATCCTTACTCTTCACACGCCGGTTTTG	C

* P = oligo used for DNA probes used in *in vitro* binding assays; M = oligo used for protein mutagenesis; C = oligo used for cloning; RT = oligo used in RealTime PCR. In P and M oligos, mutations in respect to the wild type are underlined.

Table S2 - List of strains and plasmids used in this study

Strain	Description	Reference
E. coli DH5α	*supE44 ΔlacU169 (ϕ80 lacZΔM15) hsdR17 recA1 endA1 gyrA96 thi-1 relA1*	[36]
**Plasmid**	**Description**	
pBlueScript KSII (+)	Cloning vector; Amp^R	Agilent, Santa Clara, CA, USA
PBSK-*p1227*_WT	pBlueScript KSII (+) derivative, containing a 61 bp probe corresponding to the region from 1.290.664 to 1.290.725 of the *H. pylori* G27 genome obtained by annealing of oligos p1227WT_F/ p1227WT_R, corresponding to the promoter of the *HPG27_RS06145* gene (*hp1227* according to *H. pylori* strain 26695 annotation)	This work
PBSK-*p1227*_T1F	pBlueScript KSII (+) derivative, containing a 61 bp probe corresponding to the region from 1.290.664 to 1.290.725 of the *H. pylori* G27 genome obtained by annealing of oligos p1227T1F_F/ p1227T1F_R, corresponding to the mutated promoter of the *HPG27_RS06145* gene (*hp1227* according to *H. pylori* strain 26695 annotation)	This work
PBSK-*p1227*_T2F	pBlueScript KSII (+) derivative, containing a 61 bp probe corresponding to the region from 1.290.664 to 1.290.725 of the *H. pylori* G27 genome obtained by annealing of oligos p1227T2F_F/ p1227T2F_R, corresponding to the mutated promoter of the *HPG27_RS06145* gene (*hp1227* according to *H. pylori* strain 26695 annotation)	This work
PBSK-*p1227*_T3F	pBlueScript KSII (+) derivative, containing a 61 bp probe corresponding to the region from 1.290.664 to 1.290.725 of the *H. pylori* G27 genome obtained by annealing of oligos p1227T3F_F/ p1227T3F_R, corresponding to the mutated promoter of the *HPG27_RS06145* gene (*hp1227* according to *H. pylori* strain 26695 annotation)	This work
PBSK-*p1227*_A1F	pBlueScript KSII (+) derivative, containing a 61 bp probe corresponding to the region from 1.290.664 to 1.290.725 of the *H. pylori* G27 genome obtained by annealing of oligos p1227A1F_F/ p1227 A1F_R, corresponding to the mutated promoter of the *HPG27_RS06145* gene (*hp1227* according to *H. pylori* strain 26695 annotation)	This work
PBSK-*p1227*_A2F	pBlueScript KSII (+) derivative, containing a 61 bp probe corresponding to the region from 1.290.664 to 1.290.725 of the *H. pylori* G27 genome obtained by annealing of oligos p1227A2F_F/ p1227A2F_R, corresponding to the mutated promoter of the *HPG27_RS06145* gene (*hp1227* according to *H. pylori* strain 26695 annotation)	This work
PBSK-*p1227*_GF	pBlueScript KSII (+) derivative, containing a 61 bp probe corresponding to the region from 1.290.664 to 1.290.725 of the *H. pylori* G27 genome obtained by annealing of oligos p1227GF_F/ p1227GF_R, corresponding to the mutated promoter of the *HPG27_RS06145* gene (*hp1227* according to *H. pylori* strain 26695 annotation)	This work
PBSK-*p1227*_ΔF	pBlueScript KSII (+) derivative, containing a 61 bp probe corresponding to the region from 1.290.664 to 1.290.725 of the *H. pylori* G27 genome obtained by annealing of oligos p1227ΔF_F/ p1227ΔF_R, corresponding to the mutated promoter of the *HPG27_RS06145* gene (*hp1227* according to *H. pylori* strain 26695 annotation)	This work
PBSK-*p1227*_T1S	pBlueScript KSII (+) derivative, containing a 61 bp probe corresponding to the region from 1.290.664 to 1.290.725 of the *H. pylori* G27 genome obtained by annealing of oligos p1227T1S_F/ p1227T1S _R, corresponding to the mutated promoter of the *HPG27_RS06145* gene (*hp1227* according to *H. pylori* strain 26695 annotation)	This work
PBSK-*p1227*_T2S	pBlueScript KSII (+) derivative, containing a 61 bp probe corresponding to the region from 1.290.664 to 1.290.725 of the *H. pylori* G27 genome obtained by annealing of oligos p1227T2S_F/ p1227T2S _R, corresponding to the mutated promoter of the *HPG27_RS06145* gene (*hp1227* according to *H. pylori* strain 26695 annotation)	This work
PBSK-*p1227*_T3S	pBlueScript KSII (+) derivative, containing a 61 bp probe corresponding to the region from 1.290.664 to 1.290.725 of the *H. pylori* G27 genome obtained by annealing of oligos p1227T3S_F/ p1227T3S_R, corresponding to the mutated promoter of the *HPG27_RS06145* gene (*hp1227* according to *H. pylori* strain 26695 annotation)	This work
PBSK-*p1227*_A1S	pBlueScript KSII (+) derivative, containing a 61 bp probe corresponding to the region from 1.290.664 to 1.290.725 of the *H. pylori* G27 genome obtained by annealing of oligos p1227A1S_F/ p1227A1S_R, corresponding to the mutated promoter of the *HPG27_RS06145* gene (*hp1227* according to *H. pylori* strain 26695 annotation)	This work

PBSK-*p1227*_A2S	pBlueScript KSII (+) derivative, containing a 61 bp probe corresponding to the region from 1.290.664 to 1.290.725 of the *H. pylori* G27 genome obtained by annealing of oligos p1227A2S _F/ p1227A2S_R, corresponding to the mutated promoter of the *HPG27_RS06145* gene (*hp1227* according to *H. pylori* strain 26695 annotation)	This work
PBSK-*p1227*_GS	pBlueScript KSII (+) derivative, containing a 61 bp probe corresponding to the region from 1.290.664 to 1.290.725 of the *H. pylori* G27 genome obtained by annealing of oligos p1227GS_F/ p1227GS_R, corresponding to the mutated promoter of the *HPG27_RS06145* gene (*hp1227* according to *H. pylori* strain 26695 annotation)	This work
PBSK-*p1227*_ΔS	pBlueScript KSII (+) derivative, containing a 61 bp probe corresponding to the region from 1.290.664 to 1.290.725 of the *H. pylori* G27 genome obtained by annealing of oligos p1227ΔS_F/ p1227ΔS_R, corresponding to the mutated promoter of the *HPG27_RS06145* gene (*hp1227* according to *H. pylori* strain 26695 annotation)	This work
pGEMt-Easy	Cloning vector; Amp^R	Promega, Madison, WI, USA
pGEMt-*P1227* WT	pGEMt-Easy derivative, containing a 61 bp probe corresponding to the region from 1.290.664 to 1.290.725 of *H. pylori* G27 genome. The region corresponds to the promoter of *HPG27_RS06145* gene (*hp1227* according to *H. pylori* 26695 strain annotation)	This work
pSB1075	Plasmid vector containing the 5.8 kb *Photorhabdus luminescens luxCDABE* operon cassette; Amp^R	[39]
pLux	PSB1075 derivative in which promoter *lasRI'* was deleted to generate a promoterless *lux* operon	This work
pLux-*p1227*_WT	pLux derivative containing a 61 bp DNA fragment corresponding to the *hp1227* promoter amplified by PCR with oligos p1227EcoRV_F/p1227EcoRV_R from PBSK-*p1227*_WT cloned upstream the *luxCDABE* operon	This work
pLux-*p1227*_T1F	pLux derivative containing a 61 bp DNA fragment corresponding to the mutated *hp1227* promoter amplified by PCR with oligos p1227EcoRV_F/p1227EcoRV_R from PBSK-*p1227*_T1F cloned upstream the *luxCDABE* operon	This work
pLux-*p1227*_T2F	pLux derivative containing a 61 bp DNA fragment corresponding to the mutated *hp1227* promoter amplified by PCR with oligos p1227EcoRV_F/p1227EcoRV_R from PBSK-*p1227*_T2F cloned upstream the *luxCDABE* operon	This work
pLux-*p1227*_T3F	pLux derivative containing a 61 bp DNA fragment corresponding to the mutated *hp1227* promoter amplified by PCR with oligos p1227EcoRV_F/p1227EcoRV_R from PBSK-*p1227*_T3F cloned upstream the *luxCDABE* operon	This work
pLux-*p1227*_A1F	pLux derivative containing a 61 bp DNA fragment corresponding to the mutated *hp1227* promoter amplified by PCR with oligos p1227EcoRV_F/p1227EcoRV_R from PBSK-*p1227*_A1F cloned upstream the *luxCDABE* operon	This work
pLux-*p1227*_A2F	pLux derivative containing a 61 bp DNA fragment corresponding to the mutated *hp1227* promoter amplified by PCR with oligos p1227EcoRV_F/p1227EcoRV_R from PBSK-*p1227*_A2F cloned upstream the *luxCDABE* operon	This work
pLux-*p1227*_GF	pLux derivative containing a 61 bp DNA fragment corresponding to the mutated *hp1227* promoter amplified by PCR with oligos p1227EcoRV_F/p1227EcoRV_R from PBSK-*p1227*_GF cloned upstream the *luxCDABE* operon	This work
pLux-*p1227*_ΔF	pLux derivative containing a 61 bp DNA fragment corresponding to the mutated *hp1227* promoter amplified by PCR with oligos p1227EcoRV_F/p1227EcoRV_R from PBSK-*p1227*_ΔF cloned upstream the *luxCDABE* operon	This work
pLux-*p1227*_T1S	pLux derivative containing a 61 bp DNA fragment corresponding to the mutated *hp1227* promoter amplified by PCR with oligos p1227EcoRV_F/p1227EcoRV_R from PBSK-*p1227*_T1S cloned upstream the *luxCDABE* operon	This work
pLux-*p1227*_T2S	pLux derivative containing a 61 bp DNA fragment corresponding to the mutated *hp1227* promoter amplified by PCR with oligos p1227EcoRV_F/p1227EcoRV_R from PBSK-*p1227*_T2S cloned upstream the *luxCDABE* operon	This work
pLux-*p1227*_T3S	pLux derivative containing a 61 bp DNA fragment corresponding to the mutated *hp1227* promoter amplified by PCR with oligos p1227EcoRV_F/p1227EcoRV_R from PBSK-*p1227*_T3S cloned upstream the *luxCDABE* operon	This work
pLux-*p1227*_A1S	pLux derivative containing a 61 bp DNA fragment corresponding to the mutated *hp1227* promoter amplified by PCR with oligos p1227EcoRV_F/p1227EcoRV_R from PBSK-*p1227*_A1S cloned upstream the *luxCDABE* operon	This work
pLux-*p1227*_A2S	pLux derivative containing a 61 bp DNA fragment corresponding to the mutated *hp1227* promoter amplified by PCR with oligos p1227EcoRV_F/p1227EcoRV_R from PBSK-*p1227*_A2S cloned upstream the *luxCDABE* operon	This work

pLux-*p1227*_GS	pLux derivative containing a 61 bp DNA fragment corresponding to the mutated *hp1227* promoter amplified by PCR with oligos p1227EcoRV_F/p1227EcoRV_R from PBSK-*p1227*_GS cloned upstream the *luxCDABE* operon	This work
pLux-*p1227*_ΔS	pLux derivative containing a 61 bp DNA fragment corresponding to the mutated *hp1227* promoter amplified by PCR with oligos p1227EcoRV_F/p1227EcoRV_R from PBSK-*p1227*_ ΔS cloned upstream the *luxCDABE* operon	This work
pTrcHisA	Expression vector for N-terminal 6xHis-tag cloning; Amp^R	ThermoFisher Scientific, Waltham, MA, USA
pTrc::1043	Derivative of pTrcHisA expressing the HP1043 response regulator; Amp^R	[10]
pTrc::1043_CTD	pTrcHisA derivative containing the C-terminal domain (CTD) of HP1043 (residues 119-223) amplified by PCR with oligos HP1043_CTD_NheI_F and HP1043_BamHI_R cloned in frame with the His-tag afted digestion with NheI and BamHI enzymes; Amp^R	This work

Table S3 – ΔΔG binding energy (kcal/mol) obtained from alanine scanning

	Chain A	Chain B
**I188**	-0.14	-0.66
**N189**	-0.58	-1.63
**Q190**	-0.00	-1.36
**R192**	-9.40	-19.22
**Q193**	-1.59	-12.82
**K194**	-1.96	-6.68
**D196**	2.55	1.80
**K197**	-0.89	-2.07
**T206**	-0.34	-2.92
**R208**	-3.04	-10.34
**R209**	-0.15	-3.81
